# XPC Lys939Gln polymorphism contributes to colorectal cancer susceptibility: evidence from a meta-analysis

**DOI:** 10.1186/1746-1596-9-120

**Published:** 2014-06-19

**Authors:** Qiliu Peng, Xianjun Lao, Weizhong Tang, Zhiping Chen, Ruolin Li, Xue Qin, Shan Li

**Affiliations:** 1 Department of Clinical Laboratory, First Affiliated Hospital of Guangxi Medical University, Nanning 530021, Guangxi, China; 2 Department of Anal and Colorectal Surgery, First Affiliated Hospital of Guangxi Medical University, Nanning, Guangxi, China; 3 Department of Occupational Health and Environmental Health, School of Public Health at Guangxi Medical University, Nanning, Guangxi, China; 4 Department of Medicine Research, First Affiliated Hospital of Guangxi Medical University, Nanning, Guangxi, China

**Keywords:** Colorectal cancer, XPC, Polymorphism, Meta-analysis

## Abstract

**Abstract:**

**Background**

Published studies investigating the association between *XPC* Lys939Gln polymorphism and colorectal cancer (CRC) risk reported inconclusive results. We performed a meta-analysis to derive a precise estimation of the relationship.

**Methods**

A comprehensive literature search was done in databases PubMed, EMBASE, and Cochrane library up to December 2013. The association between *XPC* Lys939Gln polymorphism and CRC risk was assessed by odds ratios (ORs) together with their 95% confidence intervals (CIs).

**Results**

Eight studies with 3,301 cases and 4,177 controls were included in the meta-analysis. We observed that the *XPC* Lys939Gln polymorphism was correlated with an increased CRC risk when all studies were pooled into the meta-analysis (Gln/lys vs. Lys/Lys: OR = 1.293, 95% CI 1.169–1.430, *P* = 0.000; Gln/Gln + Gln/lys vs. Lys/Lys: OR = 1.260, 95% CI 1.145–1.388, *P* = 0.000). In stratified analyses by ethnicity, smoking, and study quality, significant increased CRC risk was found in Asians (Gln/lys vs. Lys/Lys: OR = 1.345, 95% CI 1.187–1.523, *P* = 0.000; Gln/Gln + Gln/lys vs. Lys/Lys: OR = 1.317, 95% CI 1.170–1.484, *P* = 0.000), nonsmokers (Gln/Gln + Gln/lys vs. Lys/Lys: OR = 1.286, 95% CI 1.020–1.622, *P* = 0.033), and high quality studies. In subgroup analysis by source of control, significant increased CRC risk was found in both hospital-based studies and population-based studies. However, in subgroup analysis according to cancer location, no any significant association was detected.

**Conclusions**

This meta-analysis suggests that the *XPC* is a candidate gene for CRC susceptibility. The *XPC* Lys939Gln polymorphism may play an important role in CRC development among Asians and nonsmokers. Further large and well-designed studies are needed to confirm this association.

**Virtual Slides:**

The virtual slide(s) for this article can be found here: http://www.diagnosticpathology.diagnomx.eu/vs/1665902729125948

## Background

Colorectal cancer (CRC) is one of the most common cancers and is the third leading cause of cancer-related death worldwide [1,2]. In Europe and the USA, CRC represents one of the main causes of cancer deaths [1,3]. In Asia, CRC is the fourth leading cause of mortality by cancer, and its incidence is increasing [4,5]. In recent years, the incidence of CRC is increasing in China, which accounts for about 6.5% of total cancers in urban areas and 4.6 % in rural areas [6]. Previous epidemiological studies have identified dietary factors, such as consumption of meat, especially red meat, alcohol consumption, and cigarette smoking as possible risk factors for the development of CRC [7,8]. However, most individuals exposed to these known dietary risk factors never develop CRC while many CRC cases develop among individuals without those known risk factors, suggesting that other factors such as genetic factors also play important roles in colorectal carcinogenesis.

The xeroderma pigmentosum complementation group C (XPC) is one of the key members in the nucleotide excision repair (NER) pathway [9]. The NER pathway is the primary mechanism for removal of adducts from DNA, and thus is an important part of the cellular defense against a large variety of structurally unrelated DNA lesions. The XPC binds to HR23B and forms the XPC-HR23B complex, which is involved in the DNA damage recognition and DNA repair initiation in the NER pathway and the binding of XPC to damaged DNA is the rate-limiting step for NER [10-12]. The *XPC* gene is located at chromosome 3p25 and contains 16 exons and 15 introns. There are at least 687 reported single nucleotide polymorphisms (SNPs) in the *XPC* gene region (http://www.ncbi.nlm.nih.gov/snp/). Among all the identified SNPs, Lys939Gln polymorphism has received much attention in recent years. It is a substitution of lysine for glutamine in exon 15 of the *XPC* gene [13], and the variant 939Gln allele have been reported to correlated with reduced DNA repair activity and increased cancer risk [14-16].

Over the last two decades, several molecular epidemiological studies have evaluated the association between *XPC* Lys939Gln polymorphism and CRC risk, but the results remain controversial and inconclusive. For genetic association studies that check candidate polymorphisms, sample size is an important influencing factor for study accuracy. Small sample size might have insufficient power to explore a true association of modest effect [17], especially for complex multifactorial disease such as CRC [18]. Combining data from all eligible studies by meta-analysis has the advantage of increasing statistical power and reducing random error and obtaining precise estimates for some potential genetic associations. Therefore, in this study, we conducted a quantitative meta-analysis including all eligible studies.

## Methods

### Search strategy

We searched Pubmed, Embase and Cochrane library databases for all articles on the association between *XPC* Lys939Gln polymorphism and CRC risk using the following combined keywords: ‘xeroderma pigmentosum group C’, ‘XPC’, ‘colon cancer’, ‘rectal cancer’ and ‘colorectal cancer’. The latest search was done in December 2013, without any language restriction. Additional articles were identified through the references cited in the first series of articles selected. Articles included in the meta-analysis were in any language, with human subjects, published in the primary literature and had no obvious overlap of subjects with other studies. Among overlapping reports, only the studies with more information on origin of cases/controls were retained. The study was performed according to the proposal of Meta-analysis of Observational Studies in Epidemiology group (MOOSE) [19].

### Selection criteria

The following inclusion criteria were used for literature selection: (i) Case–control or cohort studies which evaluated the association between *XPC* Lys939Gln polymorphism and CRC risk; (ii) sufficient genotype data were presented to calculate the odds ratios (ORs) and 95% confidence intervals (95% CIs); (iii) control population did not contain malignant tumor patients. Major reasons for exclusion of studies were (i) review, or meta-analysis, or letter, or comment; (ii) duplicated studies, or studies without raw data we need; and (iii) studies that focused on HNPCC or FAP. Family–based studies of pedigrees with several affected cases per family were also excluded, because their analysis is based on linkage considerations.

### Data extraction

Two authors (Qiliu Peng and Xianjun Lao) independently reviewed and extracted data from all eligible studies. Data extracted included the first author, year of publication, country of origin, ethnicity, genotyping method, matching criteria, source of control, CRC ascertainment, total numbers of cases and controls and genotype frequencies of cases and controls. Ethnic backgrounds were categorized as Caucasian, and Asian. Smoking status (smoker or nonsmoker) was additionally recorded for stratified analysis. Smokers included current smokers and former smokers. Nonsmokers had never smoked. Cancer location was divided into colon cancer and rectum cancer and was also additionally recorded for the stratified analysis. To ensure the accuracy of the extracted information, the two authors checked the data extraction results and reached consensus on all of the data extracted. If different results were generated, they would check the data again and have a discussion to come to an agreement. A third reviewer (Weizhong Tang) was invited to the discussion if disagreement still existed.

### Quality score assessment

The quality of eligible studies was evaluated independently by two authors (Qiliu Peng and Xue Qin) according to a set of predefined criteria (Table 1) based on the scale of Thakkinstian et al. [20]. The revised criteria cover the representativeness of cases, source of controls, ascertainment of CRC, total sample size, quality control of genotyping methods, and Hardy-Weinberg equilibrium (HWE) in the control population. Disagreements were resolved by consensus. Scores ranged from 0 (lowest) to 10 (highest). Articles with scores equal to or less than 6 were considered “low-quality” studies, whereas those with scores higher than 6 were considered “high-quality” studies.

**Table 1:** Scale for quality assessment

**Criteria**	**Score**
Representativeness of cases	
Selected from cancer registry or multiple cancer center sites	2
Selected from oncology department or cancer institute	1
Selected without clearly defined sampling frame or with extensive inclusion/exclusion criteria	0
Source of controls	
Population or community based	2
Both population-based and hospital-based/healthy volunteers/blood donors	1.5
Hospital-based controls without colorectal cancer	1
Cancer-free controls without total description	0.5
Not described	0
Ascertainment of colorectal cancer	
Histological or pathological confirmation	2
Diagnosis of colorectal cancer by patient medical record	1
Not described	0
Sample size	
>1000	2
200-1000	1
<200	0
Quality control of genotyping methods	
Clearly described a different genotyping assay to confirm the data	1
Not described	0
Hardy-Weinberg equilibrium	
Hardy-Weinberg equilibrium in controls	1
Hardy-Weinberg disequilibrium in controls	0.5
No checking for Hardy-Weinberg disequilibrium	0

### Statistical analysis

Crude odds ratios (ORs) with 95% confidence intervals (CIs) were used to assess the association between the *XPC* Lys939Gln polymorphism and CRC risk. We evaluated the *XPC* Lys939Gln polymorphism and CRC risk using co-dominants (Gln/Gln vs. Lys/Lys and Gln/lys vs. Lys/Lys), recessive model (Gln/Gln vs. Gln/lys + Lys/Lys), and dominant model (Gln/Gln + Gln/lys vs. Lys/Lys). The Chi-square-based Q statistic test [21,22] was used to evaluate the between-study heterogeneity. If the result of the heterogeneity test was *P*_*Q*_ < 0.10, the pooled ORs were analyzed using the random-effects model [23]. Otherwise, the fixed-effects model [24] was selected. Subgroup analyses were performed by ethnicity, source of control, cancer location, smoking, and study quality. Sensitivity analysis was conducted by sequential omission of individual study to assess the robustness of the results. Publication bias was assessed using a Begg’s funnel plot and Egger’s regression asymmetry test [25]. If publication bias existed, the Duval and Tweedie non-parametric “trim and fill” method was used to adjust for it [26]. The distribution of the genotypes in the control population was tested for HWE using a goodness-of-fit Chi-square test. All analyses were performed using Stata software, version 12.0 (Stata Corp., College Station, TX). All *P* values were two-sided. To ensure the reliability and the accuracy of the results, two authors entered the data into the statistical software programs independently with the same results.

## Results

### Characteristics of studies

Based on the search criteria, ten studies investigating the *XPC* Lys939Gln polymorphism and CRC susceptibility were identified. Two of these articles were excluded because they did not present sufficient data for calculating OR and 95% CI [27,28]. Manual search of references cited in the eligible studies did not reveal any additional articles. As a result, a total of 8 relevant studies containing 3,301 cases and 4,177 controls were included in the meta-analysis [29-36] (Additional file 1: Figure S1). The main characteristics of these studies were listed in Table 2. Among these publications, four studies were conducted in Caucasian descent [29,30,33,36], and four were conducted in Asian descent [31,32,34,35]. Two were population–based studies [34,36] and six were hospital–based studies [29-33,35]. Three of these studies presented *XPC* Lys939Gln polymorphism genotype distributions according to smoking status (smokers and nonsmokers). The cases were histologically or pathologically confirmed as CRC in five studies [29,31,32,34,35]. Controls were mainly healthy or hospital-based populations and matched with age and gender. The genotype distributions of the controls in all of the included studies were consistent with HWE.

**Table 2:** Characteristics of eligible studies

**First author (year)**	**Country**	**Ethnicity**	**Sample size (case/control)**	**Genotyping methods**	**CRC confirmation**	**Source of control**	**Matching criteria**	**Quality scores**	**HWE(*P *** **value)**
Pardini 2008	Czech	Caucasian	532/532	PCR-RFLP	HC	HB	Age and sex	8	0.165
Gil 2012	Poland	Caucasian	133/100	PCR-RFLP	NR	HB	Region	3.5	0.803
Yue 2013	China	Asian	428/450	PCR-RFLP	PC	HB	Age and sex	6	0.964
Aizat 2013	Malaysia	Asian	255/255	PCR-RFLP	HC	HB	NR	5	0.316
Engin 2010	Turkey	Caucasian	110/116	PCR-RFLP	NR	HB	NR	5	0.642
Wu 2011	China	Asian	420/842	PCR-RFLP	HC	PB	Age, sex, smoking and BMI	9	0.639
Liu 2012	China	Asian	1028/1085	PCR-RFLP	HC	HB	Age and sex	8	0.740
Hansen 2007	Denmark	Caucasian	395/797	Endpoint reading	NR	PB	Sex	7.5	0.112

HC, Histologically confirmed; PC, Pathologically confirmed; NR Not reported; PB, Population–based; HB, Hospital–based; HWE, Hardy–Weinberg equilibrium in control population; PCR–RFLP, Polymerase chain reaction-restriction fragment length polymorphism.

### Meta-analysis

**Figure 1:**
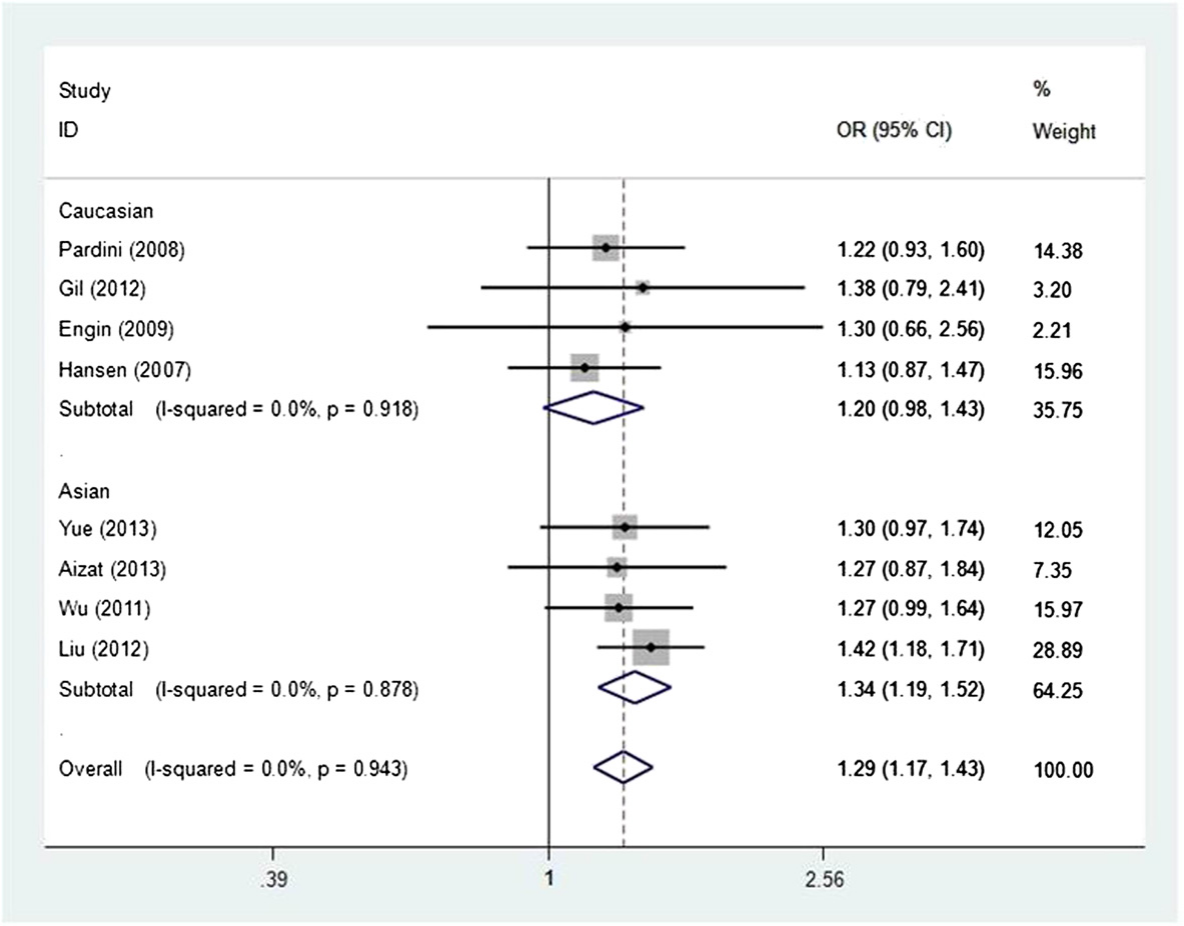
**Forest plot of subgroup analysis by ethnicity on the association between *XPC *** **Lys939Gln polymorphism and CRC risk using a fixed-effect model (Glnlys vs. LysLys).**

**Figure 2:**
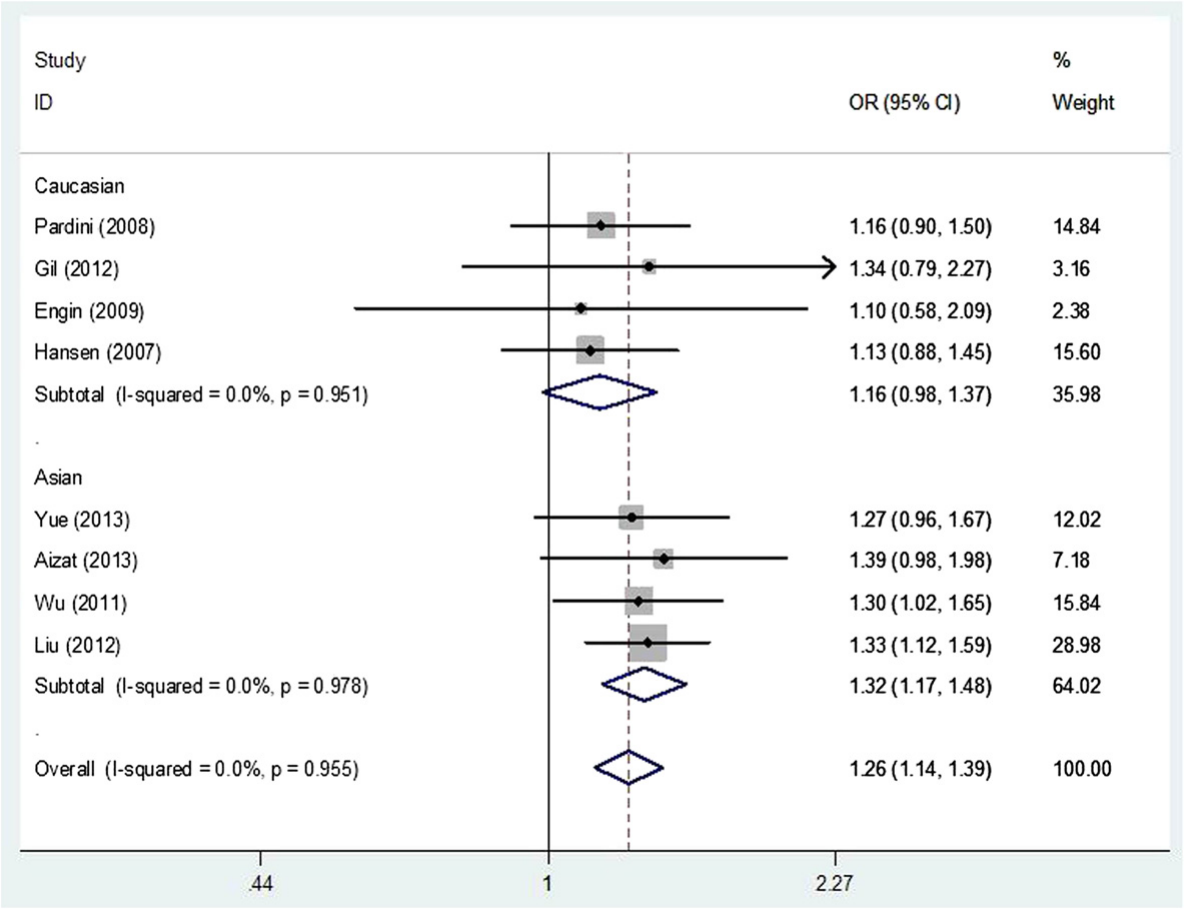
**Forest plot of subgroup analysis by ethnicity on the association between *XPC *** **Lys939Gln polymorphism and CRC risk using a fixed-effect model (dominant model GlnGln + Glnlys vs. LysLys).**

As shown in Table 3, we found that the XPC Lys939Gln polymorphism was significantly correlated with increased CRC risk when all studies were pooled into the meta-analysis (Gln/lys vs. Lys/Lys: OR = 1.293, 95% CI 1.169–1.430, *P* = 0.000; Gln/Gln + Gln/lys vs. Lys/Lys: OR = 1.260, 95% CI 1.145–1.388, *P* = 0.000). In subgroup analysis by ethnicity, significant increased CRC risk was found in Asian populations (Gln/lys vs. Lys/Lys: OR = 1.345, 95% CI 1.187–1.523, *P* = 0.000, Figure 1; Gln/Gln + Gln/lys vs. Lys/Lys: OR = 1.317, 95% CI 1.170–1.484, *P* = 0.000, Figure 2), but not in Caucasian populations. In stratified analysis according to study quality, significant increased CRC risk was found in high quality studies (Gln/lys vs. Lys/Lys: OR = 1.290, 95% CI 1.148–1.450, *P* = 0.000; Gln/Gln + Gln/lys vs. Lys/Lys: OR = 1.248, 95% CI 1.117–1.395, *P* = 0.000), but not in low quality studies. In subgroup analysis by smoking status, significant increased CRC risk was observed in nonsmokers (Gln/Gln + Gln/lys vs. Lys/Lys: OR = 1.286, 95% CI 1.020–1.622, *P* = 0.033), but not in smokers. In subgroup analysis according to source of control, significant increased CRC risk was found in both hospital-based studies (Gln/lys vs. Lys/Lys: OR = 1.335, 95% CI 1.182–1.507, *P* = 0.000; Gln/Gln + Gln/lys vs. Lys/Lys: OR = 1.282, 95% CI 1.142–1.439, *P* = 0.000) and population-based studies (Gln/lys vs. Lys/Lys: OR = 1.204, 95% CI 1.003–1.444, *P* = 0.046; Gln/Gln + Gln/lys vs. Lys/Lys: OR = 1.213, 95% CI 1.020–1.443, *P* = 0.029). However, in subgroup analysis according to cancer location, statistical significant association was not detected in both colon cancer patients and rectum cancer subjects.

**Table 3:** **Meta-analysis of *XPC *** **Lys939Gln polymorphism and colorectal cancer risk**

**Analysis**	**No. of studies**	**Homozygote**		**Heterozygote**		**Dominant model**		**Recessive model**	
		**(Gln/Gln vs. Lys/Lys)**		**(Gln/lys vs. Lys/Lys)**		**(Gln/Gln + Gln/lys vs. Lys/Lys)**		**(Gln/Gln vs. Gln/lys + Lys/Lys)**	
		**OR (95% CI)**	***P/P***_**Q**_	**OR (95% CI)**	***P/P***_**Q**_	**OR (95% CI)**	***P/P***_**Q**_	**OR (95% CI)**	***P/P***_**Q**_
Overall	8	1.134(0.979-1.315)	0.094/0.495	1.293(1.169-1.430)	0.000/0.943	1.260(1.145-1.388)	0.000/0.955	0.976(0.853-1.117)	0.722/0.211
Ethnicity									
Caucasian	4	1.038(0.818-1.318)	0.758/0.885	1.200(0.983-1.426)	0.098/0.918	1.159(0.984-1.365)	0.077/0.951	0.915(0.740-1.133)	0.417/0.644
Asian	4	1.199(0.993-1.446)	0.059/0.180	1.345(1.187-1.523)	0.000/0.878	1.317(1.170-1.484)	0.000/0.978	1.078(0.809-1.436)	0.607/0.061
Source of control									
HB	6	1.091(0.915-1.302)	0.333/0.399	1.335(1.182-1.507)	0.000/0.961	1.282(1.142-1.439)	0.000/0.945	0.922(0.785-1.082)	0.318/0.184
PB	2	1.243(0.950-1.627)	0.112/0.440	1.204(1.003-1.444)	0.046/0.528	1.213(1.020-1.443)	0.029/0.436	1.122(0.875-1.438)	0.366/0.545
Cancer location									
Colon	2	1.105(0.809-1.510)	0.528/0.327	1.278(0.983-1.601)	0.093/0.851	1.234(0.996-1.528)	0.054/0.826	0.953(0.719-1.264)	0.738/0.226
Rectum	2	1.253(0.902-1.741)	0.179/0.482	1.200(0.941-1.532)	0.142/0.471	1.217(0.967-1.531)	0.094/0.419	1.140(0.847-1.534)	0.388/0.643
Smoking									
Yes	3	1.299(0.841-2.006)	0.237/0.169	1.164(0.871-1.556)	0.305/0.825	1.118(0.900-1.389)	0.314/0.455	1.185(0.794-1.770)	0.405/0.261
No	2	1.256(0.620-2.542)	0.527/—	1.284(0.817-2.018)	0.279/—	1.286(1.020-1.622)	0.033/0.972	1.117(0.570-2.187)	0.747/—
Study quality									
High quality	4	1.093(0.920-1.299)	0.313/0.547	1.290(1.148-1.450)	0.000/0.531	1.248(1.117-1.395)	0.000/0.680	0.950(0.811-1.114)	0.530/0.310
Low quality	4	1.257(0.944-1.674)	0.118/0.304	1.201(0.963-1.563)	0.091/0.996	1.278(0.971-1.552)	0.108/0.929	1.046(0.809-1.353)	0.733/0.128

*P*_Q_ P values of Q-test for heterogeneity test. OR, odds ratio; CI, confidence intervals; HWE, Hardy–Weinberg equilibrium.

### Heterogeneity analysis

Heterogeneity between studies was estimated using the Chi-square-based Q test and the significance of which was set at *P*_*Q*_ < 0.10. There was no statistical significant heterogeneity among studies when all eligible studies were pooled into the meta-analysis. In subgroup analyses according to ethnicity, source of control, cancer location, smoking, and study quality, statistical significant heterogeneity was not observed in all subgroups (Table 3).

### Sensitivity analysis

**Figure 3:**
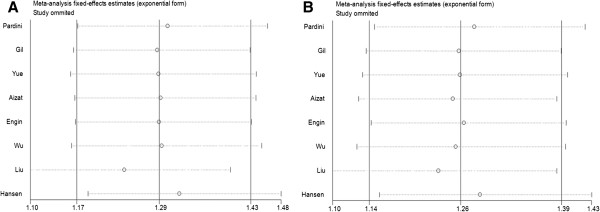
**Sensitivity analysis of *XPC *Lys939Gln polymorphism and CRC risk in the overall populations.** This figures show the influence of individual studies on the summary OR. The middle vertical axis indicates the overall OR and the two vertical axes indicate its 95% CI. Every hollow round indicates the pooled OR when the left study is omitted in this meta-analysis. The two ends of every broken line represent the 95% CI. **A** Glnlys vs. LysLys; **B** dominant model GlnGln + Glnlys vs. LysLys.

Sensitivity analysis was performed by sequential omission of individual studies. For analyses of pooling more than three individual studies, the significance of the pooled ORs was not influenced excessively by omitting any single study (Figure 3), indicating that our results were statistical robust.

### Publication bias

**Figure 4:**
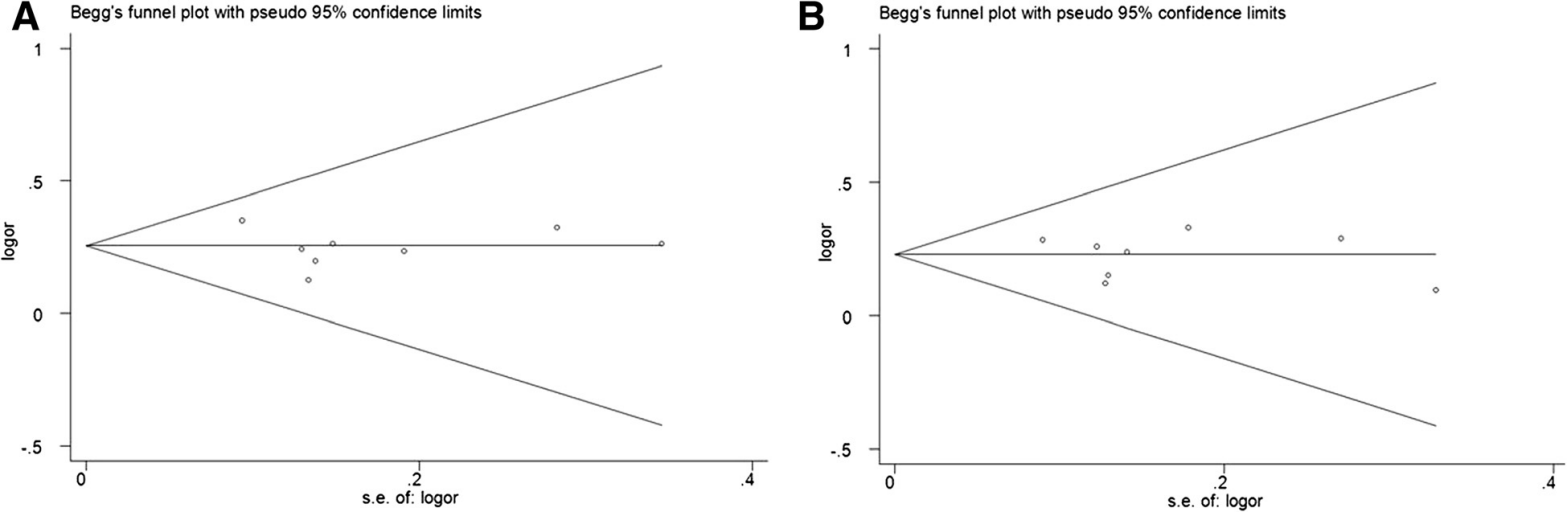
**Funnel plots for publication bias of the meta-analysis on the association between *XPC *Lys939Gln polymorphism and CRC risk in the overall populations. A** Glnlys vs. LysLys; **B** dominant model GlnGln + Glnlys vs. LysLys.

Begg’s funnel plot and Egger’s test were performed to assess the publication bias of literatures in all comparison models. The shape of the funnel plot did not reveal any evidence of obvious asymmetry (Figure 4). Then, the Egger’s test was used to provide statistical evidence of funnel plot symmetry. All the p values of Egger’s tests were more than 0.05 (P = 0.660 for GlnGln vs. LysLys; P = 0.584 for Glnlys vs. LysLys; P = 0.670 for dominant model GlnGln + Glnlys vs. LysLys; and P = 0.627 for recessive model GlnGln vs. Glnlys + LysLys), providing statistical evidence for the funnel plots’ symmetry. The results suggested that publication bias was not evident in this meta-analysis.

## Discussion

Maintenance of genomic integrity by DNA repair genes is an essential component of normal cell homeostasis necessary to cell growth, differentiation, and apoptosis [37,38]. Increasing evidence indicating that reduced DNA repair capacity, due to various DNA repair gene polymorphisms, is associated with increased risk and susceptibility to human solid tumors [16,39,40]. XPC is a key member in the NER pathway. It is involved in the recognition and initiation of the genome repair of NER pathway [10,41,42]. Polymorphisms in the *XPC* gene may alter DNA repair capacity of the NER pathway, which further play a critical role in carcinogenesis [43]. To date, several epidemiological studies have evaluated the association between *XPC* Lys939Gln polymorphism and CRC risk, but the results remain inconclusive. Meta-analysis has been recognized as an important tool to more precisely define the effect of selected genetic polymorphisms on the risk for disease and to identify potential important sources of between-study heterogeneity [2]. To derive a more precise estimation of the relationship, we performed this meta-analysis. Our meta-analysis based on 8 case–control studies suggested that the *XPC* Lys939Gln polymorphism contributes to increased CRC susceptibility.

In subgroup analysis by ethnicity, statistical significant increased CRC risk was detected in Asians. However, no significant association was found in Caucasians. Actually, it might not be uncommon for the same polymorphism playing different roles in cancer susceptibility among different ethnic populations, because cancer is a complicated multi-genetic disease, and different genetic backgrounds may contribute to the discrepancy. Nevertheless, owing to the limited number of relevant studies included in the meta-analysis, the observed association between *XPC* Lys939Gln polymorphism and CRC risk is likely to be caused by chance because study with small sample sizes may have insufficient statistical power to detect a slight effect or may have generated a fluctuated risk estimate. Currently there were only four studies on *XPC* Lys939Gln polymorphism and CRC risk for Asian populations, and Caucasian populations, respectively. Therefore, the observed results of our study should be interpreted with caution.

When stratified according to the quality score of the articles, statistical significant increased CRC risk was observed in high quality studies but not in low quality studies. The possible reason for the discrepancy may be that the existence of selection bias and recall bias in the low quality studies. In addition, genotyping methods without quality control in studies of low quality should be also considered when deciphering these inconsistent results. In subgroup analysis according to the source of control, statistical significant increased CRC risk was found in both population-based studies and hospital-based studies. However, the ORs and 95% CIs differ largely in these two subgroups. The reason may be that the hospital-based studies have a high risk of producing unreliable results because hospital-based controls may not always be truly representative of the general population. Therefore, a methodologically preferable design, such as using a proper and representative population-based high quality study, is of great value in case–control studies.

It is well established that the carcinogenesis of CRC is a result of the interaction between environmental factors and genetic background [18,44]. Besides the role of genetic variants, smoking behavior shows an important effect on the CRC susceptibility. It has been reported that smoking increased CRC risk threefold [36]. It is thought that smoking increased cancer risk due to chemicals such as hydrocarbons, arylamines, nitrosamines, and the formation of reactive oxygen species as by-products of the above compounds [45] that are known to induce bulky adducts, base damage, and DNA strand breaks. DNA repair mechanisms are paramount in correcting the changes on DNA and provide unmutated DNA while replication goes on [46]. The XPC protein plays a crucial role in repairing the DNA damage caused by tobacco smoke. Individuals with the *XPC* variant genotype may possess deficient DNA repair capability. Accordingly, the XPC protein product may be less efficient in repairing the DNA lesions induced by tobacco smoke, and thereby could enhance the susceptibility, favoring the development of CRC. Therefore, we carried out subgroup analysis according to smoking status. Our results showed an increased CRC risk in nonsmokers but not in smokers, which was inconsistent with the hypothesis above. The results should be interpreted with caution because of the limited numbers of the original studies. Therefore, further studies concerning stratification for smoking are needed to increase power for the association estimation.

Some possible limitations in this meta-analysis should be acknowledged. First, in subgroup analysis by ethnicity, the included studies regarded only Asians and Caucasians. Data concerning other ethnicities such as Africans were not found. Thus, additional studies are warranted to evaluate the effect of this functional polymorphism on CRC risk in different ethnicities, especially in Africans. Second, our results were based on unadjusted estimates. We did not perform the analysis adjusted for other covariates such as age, drinking status, environment factors, and so on, because of the unavailable original data of the eligible studies. Third, in subgroup analyses by ethnicity, cancer location, and smoking status, the sample size of the subgroups was relatively small for stratified analyses, which may lead to relatively weak power to detect the real relationship.

## Conclusions

Our meta-analysis provided a more precise estimation based on larger sample size compared with the individual studies. Our study suggested that the *XPC* is a candidate gene for CRC susceptibility. The *XPC* Lys939Gln polymorphism may play an important role in CRC development especially among Asians and nonsmokers. In order to further verify our findings, large well designed epidemiological studies are warranted.

## Abbreviations

CRC: Colorectal cancer; HWE: Hardy–Weinberg equilibrium; NER: Nucleotide excision repair; XPC: Xeroderma pigmentosum complementation group C; SNP: Single nucleotide polymorphism; OR: Odds ratio; CI: Confidence interval.

## Competing interest

All authors declare that they have no competing interest in relation to this study.

## Authors' contributions

QP, XQ performed the literature search, data extraction, statistical analysis and drafted the manuscript. QP, XL, WT, ZC, and RL participated in data extraction. QP, XQ, ZC, SL supervised the literature search, data extraction, statistical analysis and drafted the manuscript. All authors read and approved the final manuscript.

## Supplementary Material

Additional file 1 Flow diagram of included studies for this meta-analysis. Click here for file
