# Supplementary figures and images for: XPC Lys939Gln polymorphism contributes to colorectal cancer susceptibility: evidence from a meta-analysis
Source: Diagn Pathol. 2014 Jun 19;9:120. doi: 10.1186/1746-1596-9-120 (PMC4098961; doi:10.1186/1746-1596-9-120)

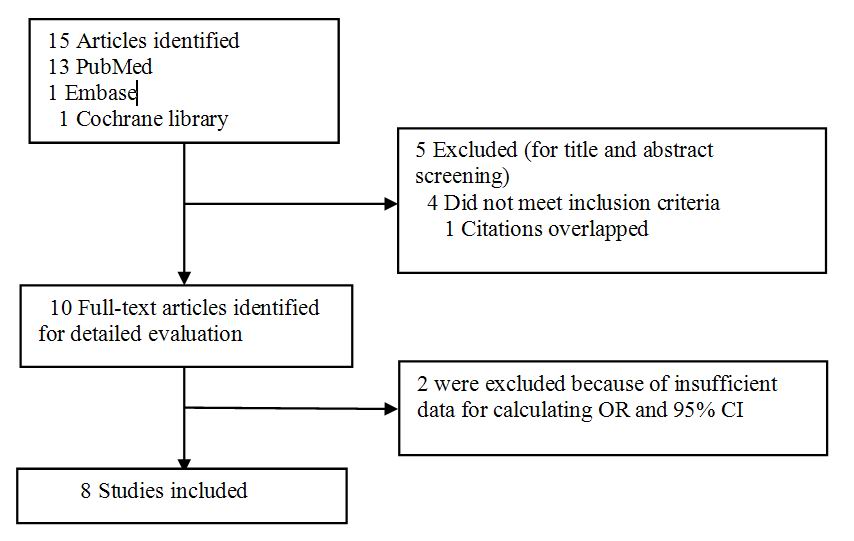

Supplement: Additional file 1 — Flow diagram of included studies for this meta-analysis. [file 1746-1596-9-120-S1.jpeg]
